# Relationships Between Hematological Variables and Bone Metabolism in Elite Female Trail Runners

**DOI:** 10.3390/healthcare14020200

**Published:** 2026-01-13

**Authors:** Marta Carrasco-Marginet, Silvia Puigarnau, Javier Espasa-Labrador, Álex Cebrián-Ponce, Fabrizio Gravina-Cognetti, Nil Piñol-Granadino, Alfredo Irurtia

**Affiliations:** 1 National Institute of Physical Education of Catalonia (INEFC), Av. De l’Estadi 12-22, 08038 Barcelona, Spain; mcarrascom@gencat.cat (M.C.-M.); spuigarnau@gencat.cat (S.P.); fgravina@gencat.cat (F.G.-C.); nilpigra@gmail.com (N.P.-G.); 2 Barcelona Research Group on Sport Sciences (GRCEIB), National Institute of Physical Education of Catalonia (INEFC), Av. De l’Estadi 12-22, 08038 Barcelona, Spain; javierespasa12@gmail.com (J.E.-L.); acebrianponce@gmail.com (Á.C.-P.); 3 Catalan School of Kinanthropometry (ECC—INEFC), National Institute of Physical Education of Catalonia (INEFC), Av. De l’Estadi 12-22, 08038 Barcelona, Spain

**Keywords:** bone mineral density, biochemical profile, hormonal and endocrine adaptations, iron metabolism, endurance athletes, low energy availability

## Abstract

**Background:** This study investigated the relationships between hematological and bone metabolism variables in 35 elite female trail runners, focusing on identifying key hematological correlates of bone health. **Methods:** Forty-four hematological variables, including biochemical, hormonal, metabolic, liver enzyme, and iron profiles, as well as complete blood count and platelet indices, were analyzed. Bone mineral density (BMD) and bone mineral content (BMC) were assessed at multiple skeletal regions via dual-energy X-ray absorptiometry (DXA). A cross-sectional design was employed, utilizing descriptive statistics, correlation analyses, and multiple linear regression to analyze the associations between hematological markers and BMC and BMD. **Results:** Significant but moderate associations were identified: magnesium consistently emerged as a negatively associated factor, particularly associated with BMC and BMD in the lumbar spine (L1–L4) and whole-body, potentially reflecting hypothesized mineral mobilization during chronic physical stress. Follicle-stimulating hormone showed positive associations with BMD, suggesting a potential protective association in bone turnover regulation. Additionally, calcium and thyroid hormones were linked to regional bone properties, highlighting site-specific skeletal vulnerabilities. **Conclusions:** These findings suggest a complex interplay between mineral homeostasis and hormonal balance that may be related to skeletal integrity in elite female trail runners. This work provides a foundation for developing evidence-based guidelines to support the health and performance of female endurance athletes. Further research is warranted to confirm these results through longitudinal evaluations.

## 1. Introduction

Elite endurance athletes, particularly female trail runners, face unique physiological challenges associated with the intense mechanical and metabolic demands of their sport [1]. These demands have been linked to adaptations and imbalances in biochemical, hormonal, hematological, and bone metabolism parameters, which are crucial for performance, recovery, and long-term health [2,3]. Specifically, the unique biomechanical stressors of trail running—characterized by significant downhill eccentric loading and multidirectional stabilization on uneven terrain—may induce distinct site-specific skeletal adaptations, necessitating a detailed analysis of regional bone mineral density (BMD). While the effects of endurance training on skeletal and metabolic health have been broadly studied, research specific to trail runners, especially female athletes prone to profiles potentially consistent with energy deficiency, menstrual dysfunction, and compromised bone health, remains limited [4].

Endurance training, characterized by high exercise volumes and prolonged energy expenditure, is strongly associated with reproductive hormone suppression, which is linked to conditions such as hypothalamic amenorrhea [5,6]. Menstrual disfunction, observed in up to 50% of female endurance athletes, is particularly prevalent in disciplines characterized by high training volumes and elevation gains, such as trail running. In this population, the chronic energy deficiency often leads to hypothalamic amenorrhea, characterized by low levels of follicle-stimulating hormone (FSH), luteinizing hormone (LH), and beta-estradiol, factors related to impaired bone remodeling and increased trabecular bone fragility [6]. Consequently, the lumbar spine (L1–L4) and other trabecular-rich regions appear particularly vulnerable, with reductions in BMD associated with a higher risk of stress fractures [7].

Beyond hormonal disruptions, endurance training has been associated with significant changes in hematological parameters, particularly affecting iron metabolism [8]. Suboptimal ferritin levels, which may be related to increased iron turnover and inadequate intake, are associated with impaired oxygen transport and collagen synthesis, potentially compromising bone health [9]. Alterations in thyroid hormones, particularly low triiodothyronine (T3) due to chronic energy deficits, are also linked to disruptions in bone remodeling by affecting osteoblastic activity [10,11].

While high-impact activities are generally associated with osteogenesis in cortical-rich regions such as the femoral neck [12], the interplay of energy deficits and hormonal imbalances may counteract these benefits, creating site-specific vulnerabilities [5]. Additional factors, such as vitamin D deficiency, present in up to one-third of endurance athletes, exacerbate these challenges by impeding calcium absorption and bone mineralization [13].

Studies on site-specific bone health, such as those assessing the reliability of calcaneal BMD measurements, have shown that female trail runners exhibit lower calcaneal BMD compared to other endurance athletes, yet higher than sedentary non-athletes [14]. However, comprehensive research exploring the associations between hematological biomarkers and bone health parameters, including bone mineral content (BMC) and BMD, across multiple skeletal regions in female trail runners, is still lacking.

This study aims to fill this gap by investigating the associations between hematological biomarkers and their relationship with BMC and BMD in elite female trail runners. Using multivariate analyses, we seek to identify key hematological factors associated with bone health that may inform potential interventions to enhance skeletal integrity and reduce injury risk in this unique athletic population.

## 2. Materials and Methods

### 2.1. Ethical Approval

The study protocol was reviewed and approved by the Ethics Committee for Clinical Research of the Catalan Sports Council on 28 July 2013 (Ethical Approval Code: 0099 S/690/2013), serving as the overarching framework for the “SUMMIT Project: Physiological Analysis in Elite Female Trail Runners”. All participants were thoroughly informed about the study’s objectives, procedures, potential benefits, and risks. Written and oral informed consent was obtained from each participant. Data collection for the present study was conducted in July 2021 (preseason testing). The study was conducted in accordance with the principles of the Declaration of Helsinki. To maintain confidentiality, all data were pseudonymized and stored in a secure, restricted-access server.

### 2.2. Participants

This cross-sectional correlational and multivariate study utilized a non-probabilistic convenience sample. Sample size estimation was performed using G*Power v3.1.9.6 to detect correlations with an effect size = 0.5 and a confidence level of α = 0.05. The statistical power (*p* = 1 − ß) was set at 0.90, resulting in a minimum required sample size of *n* = 34. The final sample consisted of 35 elite female trail runners who were members of the Spanish national team and voluntarily agreed to participate in the study. Somatic characteristics and training background of the participants are summarized in Table 1. Recruitment was facilitated through collaboration with the Spanish Federation for Mountain and Climbing Sports (FEDME).

Participants were female athletes with international competitive experience and a valid International Trail Running Association (ITRA) performance index obtained during the previous season (ITRA score). This sports performance ranking (0–1000 scale) is based on participation and race results across various competitive events during a full sports season; scores exceeding 600 denote an international standing, confirming the cohort’s elite status. Although menstrual status was not an inclusion criterion, history was documented via structured questionnaires to categorize the sample into three cohorts for exploratory analysis: naturally cycling eumenorrheic (*n* = 12), hormonal medication users (*n* = 10), and naturally cycling athletes with menstrual dysfunction (*n* = 13). To standardize hormonal influence, eumenorrheic participants were assessed during the early follicular phase (days 3–4 post-menses) [15,16]. Hormonal medication users exclusively utilized combined hormonal contraceptives (CHCs), consisting of a vaginal ring (NuvaRing^®^, Merck/MSD, Rahway, NJ, USA; *n* = 7) and low-dose oral pills (Melodene^®^, Yasminell^®^, Theramex Ireland Limited, Bray, County Wicklow, Ireland; or Loette^®^, Pfizer, New York, NY, USA; *n* = 3), with all participants reporting a long-term duration of use exceeding 8 years to ensure that analyzed variables reflect stabilized chronic physiological adaptations. While CHCs can influence endogenous hormonal profiles, preliminary comparisons, utilizing False Discovery Rate (FDR) adjustment for multiple testing, revealed no significant differences in any somatic, hematological, biochemical, or primary bone mineral metrics across the three groups (Tables S1–S4). Consequently, to maximize statistical power and given the absolute homogeneity of outcomes after statistical correction, the sample was analyzed as a single cohort, notwithstanding current stratification recommendations [17]. Exclusion was determined via self-reported data and included: metallic implants or non-removable artifacts; a history of anorexia nervosa (DSM-V criteria); use of supplements influencing body composition; and physical exercise within 48 h of testing to mitigate acute effects on tissue dynamics.

### 2.3. Procedures

Participants completed all testing procedures at the same time of day, specifically between 8:00 a.m. and 10:00 a.m., to standardize for circadian rhythm. All assessments were conducted during the sports preseason in a climate-controlled laboratory maintained at 24 °C with 50% relative humidity. Participants arrived in a fasted state (≥8 h since their last meal), having strictly refrained from all physical exercise for the previous 48 h (absolute physical rest), dressed in minimal clothing (underwear), and removed all metal objects.

Height and body mass were measured using a stadiometer (Holtain Limited^®^, Crymych, UK; range: 600–2100 mm; resolution: 1 mm) and a medical scale (Seca 710^®^, Seca Corp., Hamburg, Germany; range: 0.05–200 kg; resolution: 0.05 kg), respectively.

BMD and BMC were quantified using a Lunar Prodigy Advance^®^ scanner (GE Healthcare, Boston, MA, USA). All procedures aligned to the 2023 Official Positions of the International Society for Clinical Densitometry (ISCD). BMD results were categorized as normal, osteopenia, or osteoporosis based on T-scores and Z-scores according to the clinical criteria established by the ISCD [18]. To minimize inter-observer variance, a single experienced investigator performed and analyzed all regional scans, including the lumbar spine (L1–L4), femoral neck, femoral total, trunk, and both upper and lower limbs. Densitometric precision at this facility complies with the ISCD’s minimum acceptable reliability thresholds: ≤1.9% for the lumbar spine (L1–L4) and ≤2.5% for femoral sites. Furthermore, independent validation of the GE-Lunar Prodigy system supports the high technical reliability of these regional skeletal metrics, with reported CV values below 5% for most areas, specifically 2.5% and 3.6% for lower-limb BMD and BMC, 4.2% and 4.9% for the upper limbs, and 4.6% and 6.0% for the trunk [19]. Scans yielded an effective radiation dose of 14.24 μSv, with data processed through enCORE^®^ software, version 18.20 (GE Healthcare).

Venous blood samples were collected from the antecubital vein by a certified phlebotomist using standard aseptic techniques. Approximately 10 mL of blood was drawn into EDTA-containing tubes to prevent coagulation. This whole blood was used to measure comprehensive blood counts. An additional 10 mL of blood was collected into serum separator tubes, allowed to clot for 30 min at room temperature, and subsequently centrifuged at 1500× *g* for 10 min to obtain the serum. The serum was used for all other biochemical and hormonal analyses. Blood samples were immediately stored on ice and transported to the laboratory for analysis within 2 h to maintain sample integrity. Comprehensive blood counts were measured using an automated hematology analyzer (Sysmex XE-2100^®^, Sysmex Corporation, Kobe, Japan). Parameters included erythrocyte count, hemoglobin, hematocrit, mean corpuscular volume, mean corpuscular hemoglobin, mean corpuscular hemoglobin concentration, red cell distribution width, and erythrocyte sedimentation rate. The white blood cell count included a differential analysis (neutrophils, band neutrophils, lymphocytes, monocytes, eosinophils, and basophils), alongside platelet count and mean platelet volume. Markers of iron metabolism, including serum ferritin, transferrin, transferrin saturation, and serum iron, were assessed. Serum ferritin levels were determined using a chemiluminescent immunoassay (Abbott Architect i2000SR^®^, Abbott Diagnostics, Abbott Park, IL, USA). The biochemical and liver enzyme profiles, comprising urea, creatinine, glucose, sodium, potassium, chloride, magnesium, calcium, total cholesterol, aspartate aminotransferase (AST), alanine aminotransferase (ALT), gamma-glutamyl transferase (GGT), lactate dehydrogenase (LDH), and creatine kinase (CK), as well as serum iron and transferrin were measured using a colorimetric method (Roche Cobas 8000 c702^®^, Roche Diagnostics, Basel, Switzerland). Transferrin saturation was calculated using serum iron and transferrin levels. Hormonal profiles, including T3, beta-estradiol, and FSH, were analyzed using chemiluminescent immunoassays (Beckman Coulter Access 2^®^, Beckman Coulter, Brea, CA, USA). All assays were performed in duplicate, maintaining intra-assay coefficients of variation below 5% to ensure reliability. Equipment calibration and validation of assay procedures were conducted according to manufacturer specifications and standard laboratory protocols. Reference ranges for all physiological and biochemical parameters were based on the American Board of Internal Medicine Laboratory Test Reference Ranges (January 2024). Although elite athletes may exhibit specific physiological adaptations, these norms were utilized as the established clinical baseline given the current absence of internationally validated, sport-specific reference ranges for elite female trail runners.

### 2.4. Statistical Analysis

Descriptive statistics, including mean, standard deviation (SD), and range (minimum–maximum values), were calculated for all variables. Data normality was assessed using the Shapiro–Wilk test, and homogeneity of variances was evaluated using Levene’s test. Parametric tests, including independent *t*-tests and Pearson’s correlation coefficient, were applied to normally distributed variables. For non-normally distributed data, non-parametric alternatives, such as the Mann–Whitney U test and Spearman’s rank-order correlation, were utilized. Correlation coefficient magnitudes were interpreted based on Hopkins’ criteria [20]: trivial (0–0.09), low (0.10–0.29), moderate (0.30–0.49), large (0.50–0.69), very large (0.70–0.89), nearly perfect (0.90–0.99), and perfect (1.00). Multiple linear regression analyses were performed to identify hematological variables, acting as potential surrogates for metabolic and energy status, associated with BMC and BMD in the lumbar spine (L1–L4), femoral neck, trunk, and whole-body. For each regression model, independent variables were selected based on the significant correlations identified in the preliminary analysis. To minimize the risk of overfitting and ensure model parsimony, a forward stepwise selection method was employed, ensuring that only variables significantly contributing to the model’s variance were retained. All models were checked for multicollinearity using the variance inflation factor, with a threshold set at <10 to confirm the absence of collinearity issues. Adjusted R^2^ values were reported to indicate the proportion of variance explained by the models. Statistical significance was determined at *p* < 0.05 for all tests. For group comparisons in Supplementary Materials (Tables S1–S4), the FDR method was applied to adjust *p*-values for multiple comparisons, ensuring the robustness of the reported group homogeneity. However, due to the exploratory nature of the study and the unique elite cohort, no formal adjustment for multiple testing was applied to the primary correlational matrix to minimize the risk of Type II errors. All statistical analyses were performed using IBM SPSS Statistics^®^ 26.0 (SPSS, Inc., Chicago, IL, USA).

## 3. Results

### 3.1. Descriptive Analysis

The descriptive analysis of hematological variables, including their comparison with reference values and the prevalence of abnormal cases, is presented in Table 2.

Significant deviations from reference ranges were observed across the six categories analyzed (Table 2). In the biochemical profile, urea levels were elevated in all female athletes, while other markers, including creatinine, glucose, sodium, potassium, chloride, magnesium, and calcium, remained mostly within normal ranges. In the hormonal and endocrine profile, deviations were widespread and clinically significant: a striking 68.6% of participants had low vitamin D levels, representing the most prevalent deficiency in this profile. Additionally, 48.6% had low T3, and 28.6% showed low LH. Beta-estradiol levels were high in 17.1% of the participants, while thyroid-stimulating hormone (TSH) values were predominantly normal. For the liver enzymes and metabolic profile, 97.1% of runners had elevated lactate dehydrogenase LDH, 60.0% showed elevated creatine kinase (CK), and 40.0% had high total cholesterol, with other liver enzyme markers remaining largely within reference ranges. In the complete blood count and leukocyte profile, the cohort exhibited low neutrophil counts (100%), while 31.4% had high lymphocytes and 37.1% showed elevated monocytes. The hematological profile and iron metabolism showed low ferritin in 48.6% and reduced transferrin saturation in 28.6%, with 17.2% having low serum iron levels. In the platelet count and indices, 94.3% had elevated mean platelet volume (MPV), while platelet counts were normal in nearly all participants. These findings indicate specific deviations in metabolic, hormonal, and immune profiles relative to reference standards.

The descriptive analysis of body composition and bone metabolism indicators measured by DXA in 35 elite female trail runners is presented in Table 3. Additionally, Table 4 summarizes the prevalence of normal BMD values, osteopenia, and osteoporosis based on T-score and Z-score distributions across multiple skeletal regions, as defined by the criteria established by the ISCD.

The DXA analysis revealed that the total body mass of the female athletes was predominantly composed of lean mass, with whole-body lean body mass averaging 41.2 ± 3.1 kg, and fat mass contributing 18.9 ± 3.4%. Visceral adipose tissue levels were notably low, with a mean value of 3.0 ± 3.9 cm^2^. BMC and BMD measurements varied across skeletal regions, with whole-body BMC averaging 2206.286 ± 246.702 g and whole-body BMD at 1.096 ± 0.094 g/cm^2^ (Table 3). Table 4 indicates that, based on T-scores, 48.6% of participants exhibited osteopenia in the lumbar spine (L1–L4), with 11.4% at risk of osteoporosis. Femoral regions showed a lower prevalence of osteopenia, ranging from 5.7% in the whole body to 22.9% in the femoral total, with no cases of osteoporosis. Z-scores confirmed normal BMD values in 100% of the female athletes for femoral neck, femoral total, and whole-body measurements, while the lumbar spine (L1–L4) presented a low BMD risk in 17.1% of the cohort.

### 3.2. Correlational Analysis

The correlational analysis revealed significant relationships between hematological variables and bone parameters (BMC and BMD). DXA-derived regions showed significant correlations with both BMC and BMD, with magnesium, calcium, and T3 emerging as the most frequently associated variables (Table 5).

Magnesium exhibited large negative correlations with lumbar spine (L1–L4) BMC (r = −0.56, *p* = 0.001) and BMD (r = −0.57, *p* = 0.001), as well as large to moderate negative correlations with trunk BMC (r = −0.50, *p* = 0.002) and BMD (r = −0.46, *p* = 0.01), whole-body BMC (r = −0.46, *p* = 0.01) and BMD (r = −0.44, *p* = 0.01). Calcium displayed moderate negative correlations with lumbar spine (L1–L4) BMC (r = −0.40, *p* = 0.02), trunk BMD (r = −0.37, *p* = 0.03), and whole-body BMC (r = −0.36, *p* = 0.03) and BMD (r = −0.34, *p* = 0.05). T3 demonstrated a large positive correlation with lumbar spine (L1–L4) BMD (r = 0.51, *p* = 0.002) and moderate positive with lumbar spine (L1–L4) BMC (r = 0.40, *p* = 0.02), trunk BMC (r = 0.44, *p* = 0.01) and BMD (r = 0.39, *p* = 0.03). Other significant findings include a low positive correlation between urea and femoral neck BMC (r = 0.36, *p* = 0.03) and low negative correlations between glucose and femoral neck BMC (r = −0.38, *p* = 0.03) and BMD (r = −0.37, *p* = 0.03). MPV was low positively correlated with femoral neck BMD (r = 0.37, *p* = 0.03). Beyond these regions, additional significant correlations were exclusively observed between magnesium and lower limbs BMD (r = −0.35, *p* = 0.04) and upper limbs BMD (r = −0.34, *p* = 0.05). No other significant correlations were found between the remaining hematological variables and BMC or BMD across any of the analyzed skeletal regions.

### 3.3. Multivariate Analysis

Multiple linear regression analyses were conducted to identify hematological factors associated with BMC and BMD in the four anatomical regions that previously demonstrated significant correlations with hematological parameters in both bone parameters: (A) Lumbar spine (L1–L4): The regression models for both BMC and BMD explained substantial portions of variance, with R^2^ = 0.56 (*p* < 0.001) for BMC and R^2^ = 0.55 (*p* < 0.001) for BMD. Variables consistently associated included magnesium (BMC: β = −0.52; *p* < 0.001; BMD: β = −0.35; *p* = 0.006) and FSH (BMC: β = 0.29; *p* = 0.03; BMD: β = 0.53; *p* < 0.001). Additional factors significantly associated were calcium (BMC: β = −0.31; *p* = 0.02) and GGT (BMC: β = 0.32; *p* = 0.01) for BMC, and T3 (BMD: β = 0.28; *p* = 0.02) for BMD. (B) Femoral Neck: The variance explained was 32.0% for BMC (R^2^ = 0.32; *p* = 0.002) and 8.8% for BMD (R^2^ = 0.09; *p* = 0.046). Magnesium consistently emerged as a negative associated factor (BMC: β = −0.34; *p* = 0.046; BMD: β = −0.34; *p* = 0.05), while FSH (β = 0.39; *p* = 0.01) and glucose (β = −0.35; *p* = 0.03) were significant for BMC only. (C) Trunk: The models explained 34.9% of the variance for BMC (R^2^ = 0.35; *p* = 0.001) and 51.0% for BMD (R^2^ = 0.51; *p* < 0.001). Shared associated variables included magnesium (BMC: β = −0.50; *p* = 0.001; BMD: β = −0.44; *p* = 0.001), calcium (BMD: β = −0.30; *p* = 0.02), and FSH (BMD: β = 0.45; *p* = 0.001). Ferritin was positively associated with BMC (β = 0.37; *p* = 0.01), while eosinophils showed a positive association with BMD (β = 0.29; *p* = 0.03). (D) Whole-Body: The explained variance was 40.4% for BMC (R^2^ = 0.40; *p* = 0.001) and 52.1% for BMD (R^2^ = 0.52; *p* < 0.001). Common associated factors included magnesium (BMC: β = −0.37; *p* = 0.01; BMD: β = −0.45; *p* = 0.001), calcium (BMC: β = −0.30; *p* = 0.04; BMD: β = −0.35; *p* = 0.007), and FSH (BMC: β = 0.35; *p* = 0.02; BMD: β = 0.44; *p* = 0.001). Urea (β = 0.31; *p* = 0.02) emerged as a significant positive associated variable for BMD.

## 4. Discussion

To the best of our knowledge, this is the first study to provide novel insights into the hematological and bone densitometry imbalances that may arise from high-intensity endurance training in a unique sample of elite female trail runners.

### 4.1. Descriptive Analysis of Hematological Markers

The biochemical profile of the elite female trail runners analyzed in this study indicates a generally healthy physiological state, with adaptations consistent with endurance training [2,3]. Notably, all the female athletes exhibited elevated urea, yet creatinine and sodium remained normal, discounting acute dehydration. This systemic elevation reflects the metabolic footprint of chronic training loads and accelerated protein turnover. In this population, protein oxidation acts as a secondary energy source, increasing nitrogenous waste due to extreme caloric demands and eccentric muscle damage [3,21]. Beyond the biochemical profile, notable deviations in hormonal and endocrine biomarkers reveal critical vulnerabilities within the cohort. The strikingly high prevalence of vitamin D levels below the 30 ng/mL threshold (68.6%) constitutes a primary concern, as suboptimal levels could impair calcium homeostasis and bone mineralization, especially under persistent mechanical stress [13]. Concomitantly, the reduced levels of FSH (20.0%) and LH (28.6%) observed suggest potential hypothalamic dysfunction. These hormonal disruptions, alongside the low T3 levels found in 48.6% of the female athletes, are potentially consistent with chronic energy deficiency and the intense training stress typical of elite endurance athletes [4]. Such profiles are known to impair bone remodeling and osteoblastic activity, further exacerbating skeletal fragility [22,23].

Interestingly, although beta-estradiol levels were mostly normal, 17.1% of athletes exhibited elevated values, which may reflect potential compensatory mechanisms, such as increased androgen aromatization under profiles potentially consistent with chronic energy deficits [6]. Furthermore, reduced T4 levels in 28.6% of the cohort may reflect potential alterations in the hypothalamic–pituitary–thyroid axis [24]. Despite these disruptions, normal TSH levels in 97.1% of athletes imply central hypothyroidism as an adaptive mechanism prioritizing survival [25].

Liver enzymes and the metabolic profile indicate physiological adaptations to high training loads. Elevated LDH levels in 97.1% of athletes reflect increased muscle turnover and chronic metabolic stress [21]. Given that all participants strictly refrained from physical exercise for 48 h prior to sampling, this strikingly high prevalence suggests a state of persistent muscle-cell leakage rather than an acute response to a single training session. Similarly, elevated CK levels in 60.0% suggest persistent muscle microtrauma and possible overtraining [26]. Total cholesterol levels exceeded healthy ranges in 40.0%, potentially reflecting dietary imbalances or profiles potentially consistent with prolonged energy deficits [27]. However, liver enzymes remained within normal ranges for most athletes, emphasizing stable hepatic function despite the metabolic demands [28].

Hematological findings suggest exercise-induced immune and recovery challenges. Lower neutrophil counts in all athletes point to exercise-induced neutropenia [29], which is commonly observed in endurance athletes and may increase infection risk, although individual susceptibility varies [30]. Elevated lymphocyte percentages in 31.4% align with an adaptive immune response but could indicate immune dysregulation if prolonged [31]. These findings are consistent with known patterns of immune modulation in endurance athletes [32].

Iron metabolism showed notable deviations. While most hematological markers were within healthy ranges, 17.2% of athletes had low serum iron, and 48.6% had low ferritin levels, reflecting suboptimal iron stores despite normal hemoglobin and hematocrit. Reduced transferrin saturation in 28.6% further highlights disruptions in iron availability, potentially associated with fatigue and performance limitations [9]. These findings suggest that dietary strategies or supplementation could be beneficial in mitigating risks associated with iron-deficiency anemia [33]. However, despite the observed alterations in iron metabolism, systemic oxygen transport does not yet appear to be compromised, likely because hemoglobin and hematocrit levels remained within healthy reference ranges, ensuring adequate oxygen-carrying capacity [34].

Finally, platelet counts were generally normal, suggesting well-regulated platelet production. However, elevated MPV in 94.3% of athletes indicates platelet activation, which may reflect adaptive responses to vascular shear stress; nevertheless, chronic MPV elevation could be indicative of systemic inflammation, potentially posing vascular health risks if not properly managed [35,36].

### 4.2. Descriptive Analysis of DXA Markers

DXA-derived T-scores highlight region-specific vulnerabilities and protective adaptations in the bone health of elite female trail runners. Normal T-scores (≥−1.0 SD) ranged from 40.0% in the lumbar spine (L1–L4) to 94.3% in whole-body BMD. Osteopenia risk (−1.0 to −2.5 SD) was most frequent in the lumbar spine (L1–L4) (48.6%), followed by the femoral total (22.9%) and femoral neck (17.1%). Notably, 11.4% of athletes exhibited lumbar spine (L1–L4) T-scores indicative of osteoporosis risk (≤−2.5 SD), reflecting the region’s sensitivity to endurance training stressors [11,24]. In contrast, cortical-dominant regions like the femoral neck and whole-body showed resilience, likely due to the osteogenic effects of diverse loading stimuli inherent to the sport [37]. The lower prevalence of osteopenia observed in the femoral regions compared to the lumbar spine (L1–L4) provides clear evidence of site-specific skeletal adaptation. The femoral neck and diaphysis are subject to repetitive, high-magnitude ground reaction forces during running, which provide a potent osteogenic stimulus for bone mineral accrual in these cortical-rich areas. However, this localized adaptation contrasts sharply with the findings in the lumbar spine (L1–L4). Due to its high trabecular content and greater metabolic activity, the lumbar spine (L1–L4) is often more sensitive to systemic metabolic stressors, such as hormonal profiles potentially consistent with low energy availability or estrogen deficiency, which may override the benefits of mechanical loading. This regional divergence underscores the importance of multi-site densitometry; relying solely on femoral measurements may lead to an underestimation of systemic bone health risks in athletes whose weight-bearing sites appear well-adapted to their specific sporting demands [14]. However, hormonal imbalances, including low FSH, LH, and vitamin D deficiency, showed a potential association with increased trabecular bone vulnerability, particularly in the lumbar spine (L1–L4) [6,13].

Z-scores predominantly revealed normal BMD values (>−2.0 SD) across all athletes for the femoral neck, femoral total, and whole-body. However, 17.1% exhibited lumbar spine (L1–L4) Z-scores ≤ −2.0 SD, indicating localized risks requiring targeted interventions. The concordance between T-score and Z-score findings in the lumbar spine (L1–L4) further reinforces the clinical significance of these specific results. While T-scores provide a comparison against peak bone mass, Z-scores adjust for age-matched peers; the fact that both metrics identify significant deficits in the axial skeleton of our study population confirms a substantial clinical vulnerability that goes beyond age-related expectations. This alignment between indicators underscores the lumbar spine (L1–L4) as a critical sentinel site for detecting early bone metabolic impairment in elite female endurance athletes. In addition to hormonal and mechanical influences, other systemic factors may also contribute to the skeletal fragility observed in this study. Disruptions in iron metabolism, particularly the low ferritin levels and reduced transferrin saturation found in our cohort, may be associated with changes in bone matrix composition via impaired collagen synthesis and enzymatic activity [33].

### 4.3. Correlational Analysis: Hematological vs. Bone Metabolism (BMC and BMD)

Correlations between hematological variables (Table 5) and bone metabolism parameters (BMC and BMD) emphasize the relationship of metabolic, hormonal, and structural factors in this cohort. While statistically significant, most correlations were moderate, likely influenced by sample size and individual variability [20].

Several hematological variables correlated with BMC and BMD, reflecting systemic effects on bone metabolism. Urea demonstrated a low positive correlation with femoral neck BMC, suggesting efficient protein metabolism supporting collagen synthesis in weight-bearing regions [37]. Glucose showed low negative correlations with femoral neck BMC and BMD, potentially linked to advanced glycation end products impairing matrix integrity [38]. Magnesium exhibited moderate-to-large negative correlations with BMC and BMD across regions. Despite normal serum values in all participants, this discrepancy highlights the limitations of serum magnesium as a biomarker; it represents <1% of body stores and is homeostatically preserved at the expense of skeletal reservoirs. Under chronic physical stress or energy deficiency, the skeleton serves as a dynamic exchangeable pool to maintain extracellular magnesium levels. It is hypothesized that magnesium could be mobilized from the bone matrix to maintain systemic concentrations in response to exercise-induced losses (e.g., sweat, renal excretion). This potential sequestration from the bone might compromise mineral density, offering a plausible explanation for the negative association observed between serum levels and bone health in this elite cohort [39]. This observation highlights that in high-performance athletes, ‘normal’ serum levels may paradoxically reflect skeletal depletion rather than nutritional adequacy. Calcium also showed moderate negative correlations with whole-body BMC and BMD, potentially indicating potential mineral homeostasis dysregulation during potential energy deficits [40], although these interpretations remain speculative without the support of dynamic bone turnover biomarkers.

Thyroid hormone T3 showed moderate positive correlations with lumbar spine (L1–L4) and trunk BMC and BMD, consistent with its role in osteoblastic activity and trabecular bone remodeling [10]. MPV demonstrated low positive correlations with femoral neck BMD, suggesting vascular adaptations to mechanical stress, though chronic elevation may indicate systemic inflammation risks [41].

### 4.4. Multivariate Analysis

The multivariate regression analysis suggests interconnected associations of hematological, metabolic, and hormonal variables with bone health among elite female trail runners. The lumbar spine (L1–L4) models explained 56.4% and 55.0% of the variance in BMC and BMD, respectively. Magnesium was a negative associated factor for both, potentially reflecting its mobilization under chronic stress [39]. Calcium was negatively associated with BMC, possibly indicating skeletal calcium mobilization during potential energy deficits [40]. FSH showed positive associations with both BMC and BMD, which may reflect its potential association with bone turnover [6]. T3 and GGT also showed a positive association, suggesting potential roles in osteoblastic activity and oxidative stress adaptation, respectively [42]. For the femoral neck, the models accounted for 32.0% and 8.8% of the variance in BMC and BMD. Magnesium was a consistent negative associated factor, and glucose showed a negative association with BMC, potentially linked to collagen integrity [39]. FSH showed a positive association with BMC. The trunk models explained 34.9% and 51.0% of the variance in BMC and BMD. Magnesium and calcium were negative associated factors, while ferritin and eosinophils showed positive associations, possibly reflecting iron availability and immune–inflammatory interactions, respectively [34,40]. Finally, whole-body models explained 40.4% and 52.1% of the variance in BMC and BMD. Magnesium and calcium were consistently negative associated factors, while FSH and urea positively correlated with bone health, consistent with their potential associations in stabilizing turnover and adaptive metabolism under mechanical stress [6,37].

## 5. Conclusions

This study provides novel insights into the complex relationships between hematological biomarkers and regional bone metabolism in elite female trail runners. Our findings underscore a marked regional divergence in skeletal health: while weight-bearing cortical sites such as the femoral regions appear resilient to high-intensity training, the lumbar spine (L1–L4) exhibits significant vulnerability. This axial fragility is confirmed by the high prevalence of osteopenia and the high concordance between T-score and Z-score deficits, identifying the lumbar spine (L1–L4) as a critical sentinel site for monitoring bone health in this population. Furthermore, the identification of magnesium, FSH, and thyroid hormones as key associated factors highlights the potential interconnected relationships between mineral homeostasis and hormonal balance in maintaining skeletal integrity.

However, several limitations must be acknowledged to contextualize these results. The cross-sectional design and relatively small sample size preclude the establishment of causal relationships. Furthermore, the lack of correction for multiple testing in our primary correlational analysis may increase the risk of Type I errors; therefore, these results should be considered exploratory. Additionally, while FDR adjustments confirmed no significant differences between medicated and non-medicated cohorts, the potential for biological heterogeneity, including the broad age range, different reproductive stages, and variability in menstrual status, to mask specific hormonal associations cannot be entirely disregarded. These factors may independently be associated with bone turnover and require validation in larger, independent cohorts. Methodologically, the lack of standardized dietary intake records and direct measures of energy availability limits our ability to fully characterize the nutritional drivers of the profiles potentially consistent with observed endocrine disruptions. Additionally, the hypothesized mobilization of magnesium and calcium from the bone matrix to maintain systemic homeostasis remains speculative in the absence of dynamic bone turnover biomarkers.

Despite these limitations, this research provides a necessary foundation for future longitudinal studies. These findings stress the importance of implementing multi-site densitometry and comprehensive hematological monitoring to safeguard the long-term health of female endurance athletes. Optimizing energy availability and ensuring adequate mineral and hormonal status remain paramount strategies for refining training and recovery guidelines in the elite mountain running community.

## Figures and Tables

**Table 1:** Somatic characteristics and training background of the analyzed female trail runners.

	Mean ± SD (*n* = 35)	Range (Min–Max)
Age (years)	33.7 ± 7.5	20.8–48.8
Stature (cm)	162.7 ± 4.2	154.0–170.2
Body mass (kg)	52.7 ± 3.8	43.1–59.9
BMI (kg/m^2^)	19.9 ± 1.3	17.7–23.3
Age of first menstruation (years)	13.4 ± 2.0	9.0–20.0
Competitive level (ITRA_Score_)	656.9 ± 63.6	520.0–784.0
Age starting sport practice	25.2 ± 7.0	5.0–41.0
Previous training background (years)	7.91 ± 5.2	3.0–26.0
Training volume (hours·week^−1^)	12.9 ± 3.7	6.0–20.0

Note: BMI: Body Mass Index; ITRA_Score_: International Trail Running Association performance index (sports performance ranking on a 0–1000 scale); SD: standard deviation.

**Table 2:** Descriptive statistics and comparison with reference values for hematological variables in 35 elite female trail runners.

	Hematological Variables	Reference (Healthy Ranges) *	Mean ± SD	Range (Min–Max)	Abnormal Cases (*n*; %)	Abnormal Prevalence (>15%)	Out of Range (Lower or Higher)
Biochemical profile	Urea (mg/dL)	8.0–20.0	40.40 ± 10.49	21.0–65.0	*n* = 35; 100%	*	Higher: *n* = 35; 100%
	Creatinine (mg/dL)	0.5–1.10	0.71 ± 0.10	0.5–0.9	*n* = 0; 0%	--	--
	Glucose (mg/dL)	70.0–99.0	78.43 ± 6.72	62.0–91.0	*n* = 3; 8.6%	--	--
	Sodium (mEq/L)	136.0–145.0	141.51 ± 1.58	138.0–144.0	*n* = 0; 0%	--	--
	Potassium (mEq/L)	3.5–5.0	4.48 ± 0.35	3.9–5.6	*n* = 1; 2.9%	--	--
	Chloride (mEq/L)	98.0–106.0	104.34 ± 1.89	101.0–109.0	*n* = 4; 11.4%	--	--
	Magnesium (mg/dL)	1.6–2.6	2.15 ± 0.15	1.8–2.5	*n* = 0; 0%	--	--
	Calcium (mg/dL)	8.6–10.2	9.64 ± 0.38	8.9–10.5	*n* = 3; 8.6%	--	--
Hormonal and endocrine profile (follicular phase)	Hydroxyvitam. D (ng/mL)	30.0–60.0	26.33 ± 10.16	12.3–58.8	*n* = 24; 68.6%	*	Lower: *n* = 24; 68.6%
	FSH (mIU/mL)	2.0–9.0	5.01 ± 2.87	0.2–9.7	*n* = 7; 20.0%	*	Lower: *n* = 7; 20.0%
	LH (mIU/mL)	1.0–12.0	4.81 ± 5.04	0.1–17.6	*n* = 10; 28.6%	*	Lower: *n* = 10; 28.6%
	Beta-estradiol (pg/mL)	10.0–180.0	99.31 ± 120.71	10.0–539.1	*n* = 6; 17.1%	*	Higher: *n* = 6; 17.1%
	T3, total (ng/dL)	80.0–180.0	77.7 ± 20.6	40.0–150.0	*n* = 17; 48.6%	*	Lower: *n* = 17; 48.6%
	T4, total (µg/dL)	5.0–12.0	6.23 ± 1.50	4.3–9.9	*n* = 10; 28.6%	*	Lower: *n* = 10; 28.6%
	TSH (µIU/mL)	0.5–4.0	1.76 ± 0.83	0.7–4.7	*n* = 1; 2.9%	--	--
Liver enzymes and metabolic profile	Cholesterol, total (mg/dL)	<200.0	193.77 ± 36.16	116.0–282.0	*n* = 14; 40%	*	Higher: *n* = 14; 40.0%
	AST (U/L)	10.0–40.0	29.14 ± 8.40	18.0–48.0	*n* = 3; 8.6%	--	--
	ALT (U/L)	10.0–40.0	24.00 ± 8.44	11.0–43.0	*n* = 2; 5.7%	--	--
	GGT (U/L)	8.0–40.0	16.94 ± 6.13	8.0–31.0	*n* = 0; 0%	--	--
	LDH (U/L)	80.0–225.0	365.83 ± 77.46	158.0–546.0	*n* = 34; 97.1%	*	Higher: *n* = 34; 97.1%
	Creatine Kinase (U/L)	30.0–135.0	173.37 ± 80.31	70.0–381.0	*n* = 21; 60.0%	*	Higher: *n* = 21; 60.0%
Complete blood count and leukocyte profile	Leukocytes (10^3^/µL)	4.0–11.0	4.67 ± 0.87	3.1–6.5	*n* = 6; 17.1%	*	Lower: *n* = 6; 17.1%
	Lymphocytes (%)	30.0–45.0	36.36 ± 7.74	23.2–50.3	*n* = 11; 31.4%	*	Higher: *n* = 11; 31.4%
	Lymphocytes (10^3^/µL)	1.0–4.8.	1.67 ± 0.39	1.1–2.7	*n* = 0; 0%	--	--
	Neutrophils (%)	50.0–70.0	54.84 ± 7.77	42.0–68.0	*n* = 10; 28.6%	*	Lower: *n* = 10; 28.6%
	Band neutrophils (%)	0.0–5.0	2.60 ± 0.74	1.5–4.4	*n* = 0; 0%	--	--
	Neutrophils (10^3^/µL)	2.0–8.25	0.31 ± 0.47	0.1–1.0	*n* = 35; 100%	*	Lower: *n* = 35; 100%
	Eosinophils (%)	0.0–3.0	2.04 ± 1.50	0.4–8.1	*n* = 5; 14.3%	--	--
	Basophils (%)	0.0–1.0	0.67 ± 0.27	0.2–1.2	*n* = 5; 14.3%	--	--
	Monocytes (%)	0.0–6.0	5.78 ± 1.27	3.9–9.2	*n* = 13; 37.1%	*	Higher: *n* = 13; 37.1%
Hematological profile and Iron metabolism	Erythrocyte count (10^6^/µL)	4.2–5.9	4.48 ± 0.33	3.7–5.0	*n* = 5; 14.3%	--	--
	Hemoglobin (g/dL)	12.0–16.0	13.40 ± 0.84	11.9–15.3	*n* = 1; 2.9%	--	--
	Hematocrit (%)	37.0–47.0	39.88 ± 2.10	35.8–45.6	*n* = 3; 8.6%	--	--
	Mean corp. volume (fL)	80.0–98.0	89.31 ± 4.43	78.1–99.9	*n* = 2; 5.8%	--	--
	Mean corp. hemogl. (pg)	28.0–32.0	30.00 ± 1.62	25.6–33.2	*n* = 5; 14.3%	--	--
	Mean corp. hem co. (g/dL)	33.0–36.0	34.43 ± 0.93	32.3–36.5	*n* = 4; 11.4%	--	--
	Red cell distrib. width (%)	9.0–14.5	13.23 ± 0.72	12.1–15.0	*n* = 2; 5.7%	--	--
	Serum Iron (µg/dL)	50.0–150.0	95.63 ± 37.48	33.0–160.0	*n* = 6; 17.2%	*	Lower: *n* = 6; 17.2%
	Ferritin (ng/mL)	24.0–307.0	28.00 ± 15.16	11.0–78.0	*n* = 17; 48.6%	*	Lower: *n* = 17; 48.6%
	Transferrin (mg/dL)	200.0–400.0	290.57 ± 40.39	234.0–433.0	*n* = 1; 2.9%	--	--
	Transferrin satur. (%)	20.0–50.0	26.15 ± 9.99	7.1–44.6	*n* = 10; 28.6%	*	Lower: *n* = 10; 28.6%
	Erytr. sedim. rate (mm/h)	0.0–20.0	10.00 ± 7.44	1.0–27.0	*n* = 3; 8.6%	--	--
Platelet count and indices	Platelet count (10^3^/µL)	150.0–450.0	217.37 ± 53.19	137.0–405.0	*n* = 2; 5.7%	--	--
	Mean platelet vol. (fL)	7.0–9.0	10.14 ± 0.94	8.9–12.7	*n* = 33; 94.3%	*	Higher: *n* = 33; 94.3%

Note: FSH: Follicle-Stimulating Hormone; LH: Luteinizing Hormone; T3: Triiodothyronine; T4: Thyroxine; TSH: Thyroid-Stimulating Hormone; AST: Aspartate Aminotransferase; ALT: Alanine Aminotransferase; GGT: Gamma-Glutamyl Transferase; LDH: Lactate Dehydrogenase; SD: standard deviation; * Reference ranges from American Board of Internal Medicine Laboratory Test Reference Ranges—January 2024.

**Table 3:** Body composition and bone metabolism indicators measured by DXA in 35 elite female trail runners.

	DXA Indicators	Mean ± SD	Range (Min–Max)
Body mass (kg)	Whole-body	53.0 ± 3.6	43.6–60.5
	Upper limbs	5.2 ± 0.5	4.3–6.3
	Trunk	24.9 ± 1.8	20.5–29.2
	Lower limbs	18.8 ± 1.7	14.5–22.0
Lean body mass (kg)	Whole-body	41.2 ± 3.1	36.8–49.8
	Upper limbs	3.9 ± 0.5	3.1–5.3
	Trunk	20.8 ± 1.8	18.3–26.1
	Lower limbs	13.6 ± 1.2	11.9–16.4
Fat mass (%)	Whole-body	18.9 ± 3.4	11.7–24.9
	Upper limbs	21.7 ± 5.5	12.2–32.0
	Trunk	14.3 ± 3.9	8.3–23.9
	Lower limbs	23.9 ± 3.9	13.8–29.9
Visceral adipose tissue (cm^2^)	VAT	3.0 ± 3.9	0.1–14.0
Bone mineral content (g)	Whole-body	2206.286 ± 246.702	1840.000 ± 2838.000
	Upper limbs	272.743 ± 32.740	209.000 ± 341.000
	Trunk	593.200 ± 96.582	443.000 ± 842.000
	Lower limbs	831.029 ± 82.016	702.000 ± 1054.000
	Lumbar Spine (L1–L4)	56.031 ± 10.045	35.700 ± 81.980
	Femoral neck	4.503 ± 0.580	3.460 ± 6.000
	Femoral total	29.521 ± 4.111	23.300 ± 38.710
Bone mineral density (g/cm^2^)	Whole-body	1.096 ± 0.094	0.950 ± 1.381
	Upper limbs	0.643 ± 0.055	0.542 ± 0.764
	Trunk	0.870 ± 0.098	0.715 ± 1.155
	Lower limbs	1.198 ± 0.089	1.032 ± 1.408
	Lumbar Spine (L1–L4)	1.051 ± 0.164	0.798 ± 1.583
	Femoral neck	0.965 ± 0.123	0.726 ± 1.252
	Femoral total	1.004 ± 0.130	0.755 ± 1.291

Note: DXA: Dual-Energy X-ray Absorptiometry; SD: Standard Deviation.

**Table 4:** Bone mineral density (BMD) T-Score and Z-Score distributions among 35 female trail runners.

		Normal Values (≥−1.0 SD)	Osteopenia Risk (−1.0 to −2.5 SD)	Osteoporosis Risk (≤−2.5 SD)
BMD: T-score	Lumbar spine (L1–L4)	*n* = 14; 40.0%	*n* = 17; 48.6%	*n* = 4; 11.4%
	Femoral neck	*n* = 29; 82.9%	*n* = 6; 17.1%	*n* = 0; 0%
	Femoral total	*n* = 27; 77.1%	*n* = 8; 22.9%	*n* = 0; 0%
	Whole-body	*n* = 33; 94.3%	*n* = 2; 5.7%	*n* = 0; 0%
		**Normal Values (>−2.0)**	**Risk of Low BMD (≤−2.0)**	
BMD: Z-score	Lumbar spine (L1–L4)	*n* = 29; 82.9%	*n* = 6; 17.1%	--
	Femoral neck	*n* = 35; 100%	*n* = 0; 0%	--
	Femoral total	*n* = 35; 100%	*n* = 0; 0%	--
	Whole-body	*n* = 35; 100%	*n* = 0; 0%	--

Note: BMD: Bone Mineral Density; SD: Standard Deviation.

**Table 5:** Correlational analysis between hematological and BMC and BMD anatomical regions.

	Lumbar Spine (L1–L4)		Femoral Neck		Trunk		Whole-Body	
	BMC	BMD *	BMC	BMD	BMC	BMD	BMC	BMD
	*r* (*p*)	*r* (*p*)	*r* (*p*)	*r* (*p*)	*r* (*p*)	*r* (*p*)	*r* (*p*)	*r* (*p*)
Urea (mg/dL)	--	--	0.36 (0.03)	--	--	--	--	--
Glucose (mg/dL)	--	--	−0.38 (0.03)	−0.37 (0.03)	--	--	--	--
Magnesium (mmol/L) *	−0.56 (<0.01)	−0.57 (<0.01)	--	−0.42 (0.01)	−0.50 (<0.01)	−0.46 (0.01)	−0.46 (0.01)	−0.44 (0.01)
Calcium (mg/dL)	−0.40 (0.02)	--	--	--	--	−0.37 (0.03)	−0.36 (0.03)	−0.34 (0.05)
T3 (ng/mL) *	0.40 (0.02)	0.51 (<0.01)	--	--	0.44 (0.01)	0.39 (0.03)	--	--
Mean platelet volume (fL) *	--	--	--	0.37 (0.03)	--	--	--	--

Note: BMC: Bone Mineral Content; BMD: Bone Mineral Density; T3: Triiodothyronine; r: correlation coefficient (Pearson or Spearman); * Variables with non-normal distribution (Spearman applied); *p*: statistical significance set at *p* < 0.05.

## Data Availability

The data used in this study correspond to elite athletes from Spain and include potentially identifiable information related to age, height, weight, performance scores, sports rankings, and other sensitive health data. Although all data have been handled with the utmost confidentiality and in accordance with ethical and data protection regulations, the specific nature of the sample could allow for indirect identification of participants. For this reason, the dataset is not publicly available. However, the data can be accessed upon reasonable request to the corresponding author, provided that the request complies with relevant ethical and confidentiality requirements.

## Supplementary Materials

The following Supporting Information can be downloaded at: https://www.mdpi.com/article/10.3390/healthcare14020200/s1. Table S1: Statistical differences in somatic characteristics and training background by menstrual cycle status in 35 elite female trail runners; Table S2: Statistical differences in hematological and biochemical variables by menstrual cycle status in 35 elite female trail runners; Table S3: Statistical differences in body composition and bone metabolism indicators measured by DXA across menstrual cycle status in 35 elite female trail runners; Table S4: Statistical differences in bone mineral density (BMD) T-Score and Z-Score distributions by menstrual cycle status in 35 elite female trail runners.

## Abbreviations

The following abbreviations are used in this manuscript:

BMD	Bone Mineral Density
BMC	Bone Mineral Content
DXA	Dual-Energy X-ray Absorptiometry
BMI	Body Mass Index
FSH	Follicle-Stimulating Hormone
LH	Luteinizing Hormone
T3	Triiodothyronine
T4	Thyroxine
TSH	Thyroid-Stimulating Hormone
AST	Aspartate Aminotransferase
ALT	Alanine Aminotransferase
GGT	Gamma-Glutamyl Transferase
LDH	Lactate Dehydrogenase
CK	Creatine Kinase
MPV	Mean Platelet Volume
VAT	Visceral Adipose Tissue
ITRA	International Trail Running Association
ISCD	International Society for Clinical Densitometry
FEDME	Federación Española de Deportes de Montaña y Escalada
