# Supplementary material for: Relationships Between Hematological Variables and Bone Metabolism in Elite Female Trail Runners
Source: Healthcare (Basel). 2026 Jan 13;14(2):200. doi: 10.3390/healthcare14020200 (PMC12841380; doi:10.3390/healthcare14020200)
Supplement: Supplementary file 1 [file healthcare-14-00200-s001.zip › healthcare-4032349-supplementary.pdf]

Table S1. Statistical differences in somatic characteristics and training background by menstrual cycle status in 35 elite female trail runners.

	Amenorrheic, not medicated (n = 13)	Eumenorrheic (n = 12)	Hormonal contraceptive user (n = 10)	*p* value
Age (years)	32.67 ± 9.34	37.09 ± 6.52	30.80 ± 4.30	0.68
Stature (cm)	161.33 ± 5.10	162.95 ± 2.99	164.26 ± 3.78	0.73
Body mass (kg)	51.39 ± 4.62	54.26 ± 2.55	52.69 ± 3.53	0.68
BMI (kg/m^2)	19.75 ± 1.65	20.36 ± 0.94	19.53 ± 1.12	0.73
Age of first menstruation (years)	13.54 ± 1.20	12.75 ± 1.06	14.00 ± 3.27	0.76
Competitive level (ITRA$_{Points}$)	654.08 ± 78.18	663.75 ± 36.68	652.40 ± 73.49	0.96
Age starting sport practice	25.62 ± 8.26	27.17 ± 4.99	22.40 ± 7.21	0.73
Previous training background (years)	7.05 ± 3.92	9.93 ± 4.95	8.40 ± 6.57	0.76
Training volume (hours·week^{-1})	13.46 ± 3.43	12.67 ± 3.06	12.30 ± 4.79	0.92

BMI: Body Mass Index; ITRA: International Trail Running Association. *p*-values adjusted for multiple comparisons using the False Discovery Rate (FDR) method. No significant differences were observed across groups ($p > 0.05$).

Table S2. Statistical differences in hematological and biochemical variables by menstrual cycle status in 35 elite female trail runners.

	Hematological variables	Amenorrheic, not medicated (n = 13)	Eumenorrheic (n = 12)	Hormonal contraceptive user (n = 10)	*p* value
Biochemical profile	Urea (mg/dL)	43.77 ± 11.61	37.17 ± 9.05	39.90 ± 10.26	0.73
	Creatinine (mg/dL)	0.73 ± 0.09	0.64 ± 0.09	0.76 ± 0.08	0.35
	Glucose (mg/dL)	77.62 ± 7.67	80.67 ± 5.85	76.80 ± 6.30	0.76
	Sodium (mEq/L)	142.08 ± 1.32	141.67 ± 1.23	140.60 ± 1.96	0.64
	Potassium (mEq/L)	4.53 ± 0.40	4.50 ± 0.28	4.38 ± 0.37	0.89
	Chloride (mEq/L)	104.08 ± 2.47	104.83 ± 1.27	104.10 ± 1.73	0.87
	Magnesium (mg/dL)	2.17 ± 0.14	2.17 ± 0.16	2.09 ± 0.15	0.76
	Calcium (mg/dL)	9.76 ± 0.46	9.58 ± 0.35	9.56 ± 0.29	0.76
Hormonal and endocrine profile (follicular phase)	Hydroxyvitam. D (ng/mL)	31.60 ± 15.96	21.98 ± 6.45	27.70 ± 12.56	0.68
	FSH (mIU/mL)	13.28 ± 27.95	5.39 ± 2.01	3.84 ± 3.48	0.76
	LH (mIU/mL)	9.45 ± 13.93	4.95 ± 3.53	2.37 ± 2.55	0.68
	Beta-estradiol (pg/mL)	85.69 ± 105.53	159.62 ± 152.08	45.55 ± 58.52	0.64
	T3, total (ng /dL)	0.75 ± 0.19	0.79 ± 0.12	0.79 ± 0.30	0.96
	T4, total (µg/dL)	6.08 ± 1.00	5.59 ± 1.14	7.19 ± 2.00	0.64
	TSH (µIU/mL)	1.70 ± 0.70	1.83 ± 0.68	1.75 ± 1.16	0.97
Liver enzymes and metabolic profile	Cholesterol, total (mg/dL)	184.31 ± 28.71	187.58 ± 41.89	213.50 ± 32.91	0.68
	AST (U/L)	36.69 ± 32.24	25.50 ± 5.55	33.10 ± 10.04	0.76
	ALT (U/L)	26.23 ± 15.79	20.83 ± 5.67	27.90 ± 9.33	0.74
	GGT (U/L)	15.54 ± 8.30	20.83 ± 7.26	15.50 ± 2.64	0.68
	LDH (U/L)	387.69 ± 111.71	338.00 ± 40.34	403.80 ± 83.38	0.68
	Creatine Kinase (U/L)	238.00 ± 223.66	132.50 ± 46.16	208.40 ± 102.76	0.73
Complete blood count and leukocyte profile	Leukocytes (10^3/µL)	4.56 ± 0.81	5.00 ± 1.01	4.40 ± 0.71	0.73
	Lymphocytes (%)	36.42 ± 6.75	36.28 ± 8.23	36.38 ± 9.11	1.00
	Lymphocytes (10^3/µL)	1.65 ± 0.36	1.74 ± 0.30	1.62 ± 0.55	0.92
	Neutrophils (%)	55.15 ± 7.04	54.61 ± 8.89	54.72 ± 8.09	1.00
	Band neutrophils (%)	2.54 ± 0.65	2.82 ± 1.00	2.41 ± 0.44	0.78
	Neutrophils (10^3/µL)	0.23 ± 0.44	0.42 ± 0.51	0.30 ± 0.48	0.91
	Eosinophils (%)	1.89 ± 1.10	1.67 ± 1.11	2.67 ± 2.17	0.73
	Basophils (%)	0.69 ± 0.28	0.61 ± 0.22	0.70 ± 0.33	0.92
	Monocytes (%)	5.62 ± 1.54	6.42 ± 1.09	5.23 ± 0.76	0.64
Hematological profile and Iron metabolism	Erythrocyte count (10^6/µL)	4.45 ± 0.29	4.61 ± 0.23	4.37 ± 0.45	0.73
	Hemoglobin (g/dL)	13.33 ± 0.84	13.83 ± 0.75	12.97 ± 0.75	0.64
	Hematocrit (%)	39.42 ± 1.94	41.21 ± 2.05	38.89 ± 1.64	0.50
	Mean corp. volume (fL)	88.86 ± 3.82	89.56 ± 3.18	89.61 ± 6.47	0.96
	Mean corp. hemogl.. (pg)	30.05 ± 1.62	30.08 ± 1.05	29.85 ± 2.24	0.97
	Mean corp. hem co. (g/dL)	34.75 ± 1.04	34.42 ± 0.77	34.02 ± 0.88	0.68
	Red cell distrib. width (%)	13.25 ± 0.75	13.01 ± 0.44	13.49 ± 0.91	0.73
	Serum Iron (µg/dL)	82.08 ± 32.74	94.33 ± 35.06	114.80 ± 41.23	0.68
	Ferritin (ng/mL)	26.23 ± 11.84	29.83 ± 14.65	28.10 ± 20.25	0.96
	Transferrin (mg/dL)	284.46 ± 24.84	275.50 ± 24.59	316.60 ± 59.08	0.64
	Transferrin satur. (%)	22.52 ± 8.26	27.06 ± 9.56	29.76 ± 11.82	0.73
	Erytr. sedim. rate (mm/h)	11.15 ± 6.54	5.08 ± 2.64	14.40 ± 9.37	0.35
Platelet count and indices	Platelet count (10^3/µL)	209.23 ± 48.48	219.83 ± 35.71	225.00 ± 76.33	0.92
	Mean platelet vol. (fL)	10.15 ± 0.86	10.17 ± 0.98	10.11 ± 1.10	1.00

FSH: Follicle-Stimulating Hormone; LH: Luteinizing Hormone; T3: Triiodothyronine; T4: Thyroxine; TSH: Thyroid-Stimulating Hormone; AST: Aspartate Aminotransferase; ALT: Alanine Aminotransferase; GGT: Gamma-Glutamyl Transferase; LDH: Lactate Dehydrogenase. *p*-values adjusted for multiple comparisons using the False Discovery Rate (FDR) method. No significant differences were observed across groups ($p > 0.05$).

Table S3. Statistical differences in body composition and bone metabolism indicators measured by DXA across menstrual cycle status in 35 elite female trail runners.

	DXA indicators	Amenorrheic, not medicated (n = 13)	Eumenorrheic (n = 12)	Hormonal contraceptive user (n = 10)	*p* value
Body mass (kg)	Whole-body	51.823 ± 4.649	54.200 ± 2.002	53.060 ± 3.483	0.73
	Upper limbs	5.146 ± 0.491	5.325 ± 0.461	5.270 ± 0.602	0.92
	Trunk	24.354 ± 2.285	25.158 ± 0.848	25.370 ± 1.922	0.76
	Lower limbs	18.323 ± 1.939	19.642 ± 0.984	18.360 ± 1.765	0.68
Lean body mass (kg)	Whole-body	42.184 ± 3.587	43.880 ± 2.455	44.289 ± 3.565	0.73
	Upper limbs	4.033 ± 0.474	4.208 ± 0.378	4.306 ± 0.600	0.76
	Trunk	20.833 ± 1.903	21.325 ± 1.154	22.313 ± 2.300	0.68
	Lower limbs	14.050 ± 1.350	15.023 ± 1.004	14.353 ± 1.216	0.68
Fat mass (%)	Whole-body	19.315 ± 3.099	19.858 ± 3.197	17.250 ± 3.778	0.68
	Upper limbs	23.038 ± 5.944	22.075 ± 4.897	19.450 ± 5.350	0.73
	Trunk	14.638 ± 3.823	15.583 ± 3.950	12.460 ± 3.537	0.68
	Lower limbs	24.246 ± 3.437	24.583 ± 3.425	22.620 ± 4.828	0.82
Visceral adipose tissue (cm^2)	VAT	2.000 ± 2.309	5.417 ± 4.999	1.400 ± 2.547	0.50
Bone mineral content (g)	Whole body	2151.000 ± 340.570	2241.250 ± 203.183	2236.200 ± 133.326	0.90
	Upper limbs	265.615 ± 37.387	273.083 ± 32.146	281.600 ± 27.605	0.87
	Trunk	565.615 ± 124.226	609.167 ± 77.022	609.900 ± 75.260	0.79
	Lower limbs	817.385 ± 108.222	848.667 ± 59.004	827.600 ± 69.594	0.91
	Lumbar Spine L1-L4	53.226 ± 13.720	58.544 ± 6.956	56.660 ± 7.131	0.78
	Femoral neck	4.473 ± 0.675	4.577 ± 0.470	4.455 ± 0.618	0.96
	Femoral total	29.215 ± 4.798	30.566 ± 4.088	28.664 ± 3.212	0.87
Bone mineral density (g/cm^2)	Whole body	1.088 ± 0.117	1.113 ± 0.091	1.085 ± 0.065	0.92
	Upper limbs	0.640 ± 0.059	0.649 ± 0.062	0.640 ± 0.045	0.96
	Trunk	0.852 ± 0.126	0.888 ± 0.083	0.872 ± 0.076	0.92
	Lower limbs	1.196 ± 0.083	1.225 ± 0.099	1.169 ± 0.083	0.76
	Lumbar Spine L1-L4	1.039 ± 0.214	1.081 ± 0.125	1.032 ± 0.141	0.92
	Femoral neck	0.965 ± 0.124	0.976 ± 0.132	0.953 ± 0.122	0.96
	Femoral total	1.008 ± 0.135	1.029 ± 0.145	0.967 ± 0.106	0.87

DXA: Dual-energy X-ray absorptiometry. *p*-values adjusted for multiple comparisons using the False Discovery Rate (FDR) method. No significant differences were observed across groups ($p > 0.05$).

Table S4. Statistical differences in bone mineral density (BMD) T-Score and Z-Score distributions by menstrual cycle status in 35 elite female trail runners.

		Amenorrheic, not medicated (n = 13)	Eumenorrheic (n = 12)	Hormonal contraceptive user (n = 10)	*p* value
BMD: T-score	Lumbar spine L1-L4	-1.262 ± 1.738	-0.908 ± 1.024	-1.300 ± 1.142	0.92
	Femoral neck	-0.138 ± 1.044	-0.033 ± 1.114	-0.230 ± 1.017	0.96
	Femoral total	0.077 ± 1.123	0.242 ± 1.196	-0.270 ± 0.892	0.87
	Whole-body	0.077 ± 1.171	0.317 ± 0.896	0.040 ± 0.640	0.92
BMD: Z-score	Lumbar spine L1-L4	-0.892 ± 1.837	-0.600 ± 0.995	-1.060 ± 1.138	0.92
	Femoral neck	0.108 ± 1.094	0.242 ± 1.133	-0.030 ± 0.984	0.96
	Femoral total	0.300 ± 1.147	0.450 ± 1.218	-0.090 ± 0.844	0.87
	Whole-body	0.677 ± 1.096	0.808 ± 0.877	0.570 ± 0.693	0.96

BMD: Bone Mineral Density. *p*-values adjusted for multiple comparisons using the False Discovery Rate (FDR) method. No significant differences were observed across groups ($p > 0.05$).
